# Cytogenetic and genomic analysis of a patient with turner syndrome and t(2;12): a case report

**DOI:** 10.1186/s13039-020-00515-0

**Published:** 2020-11-13

**Authors:** Paola E. Leone, Verónica Yumiceba, Ariana Jijón-Vergara, Andy Pérez-Villa, Isaac Armendáriz-Castillo, Jennyfer M. García-Cárdenas, Santiago Guerrero, Patricia Guevara-Ramírez, Andrés López-Cortés, Ana K. Zambrano, Jesús M. Hernández-Rivas, Juan Luis García, César Paz-y-Miño

**Affiliations:** 1 Centro de Investigación Genética y Genómica, Facultad de Ciencias de la Salud Eugenio Espejo, Universidad UTE, Av. Mariscal Sucre y Av. Mariana de Jesús, Sede Occidental, Bloque I, 2 Floor, 170129 Quito, Ecuador; 2 Servicio de Hematología, Hospital Universitario de Salamanca, Universidad de Salamanca, Salamanca, Spain; 3 Molecular Medicine Unit, Department of Medicine, Biomedical Research Institute of Salamanca (IBSAL), Salamanca, Spain; 4 Institute of Molecular and Cellular Biology of Cancer (IBMCC), University of Salamanca, Salamanca, Spain

**Keywords:** Turner syndrome, Reciprocal translocation, Cytogenetics, Genetic mapping arrays, FISH

## Abstract

**Background:**

Turner syndrome is a genetic disorder that affects women. It is caused by an absent or incomplete X chromosome, which can be presented in mosaicism or not. There are 12 cases of Turner syndrome patients who present structural alterations in autosomal chromosomes.

**Case presentation:**

The present case report describes a patient with a reciprocal, maternally inherited translocation between chromosomes 2 and 12 with a mosaicism of X monosomy 45,X,t(2;12)(p13;q24)[95]/46,XX,t(2;12)(p13;q24)[5]. Through genetic mapping arrays, altered genes in the patient were determined within the 23 chromosome pairs. These genes were associated with the patient’s clinical features using a bioinformatics tool.

**Conclusion:**

To our knowledge, this is the first case in which a translocation (2;12) is reported in a patient with Turner syndrome and confirmed by conventional cytogenetics, FISH and molecular genetics. Clinical features of our patient are closely related with the loss of one X chromosome, however mild intellectual disability can be likely explained by autosomal genes. The presence of familial translocations was a common finding, thus emphasizing the need for familiar testing for further genetic counselling.

**Electronic supplementary material:**

The online version of this article (10.1186/s13039-020-00515-0) contains supplementary material, which is available to authorized users.

## Background

Turner syndrome (TS) is a chromosomal disorder caused by a complete or partial monosomy of the X chromosome. It affects about 1 out of every 2500 newborn girls. This is the only genetic disorders where the absence of a whole chromosome is compatible with life [1]. This condition presents several cytogenetic variants, being the monosomy 45,X the most frequent (45–55%), followed by structural changes in a X chromosome (25–30%) as isochromosome of the long arm, isochromosome mosaic, deletions and ring chromosomes [1, 2]. In Ecuador, X chromosome monosomy represents 40.42%, TS mosaic accounts for 25.3% and structural alterations of X chromosome appeared in 7.9% of TS patients [3].

TS complex phenotype is characterized by growth failure, delayed puberty, primary amenorrhea, gonadal dysgenesis, ovarian insufficiency, alopecia, hirsutism, low extremity lymphedema, nail dysplasia or hypoplasia, vitiligo, short and webbed neck (*pterygium colli*), low posterior hairline at the back of the neck, wide-spaced nipples, inverted nipples, shield shaped thorax, low set ears and long term complications such as kidney, cardiovascular, and ophthalmological problems [4]. Around two-thirds of the girls with TS have the maternal X chromosome, ruling out the possibility meiotic error in elder mothers [5].

For TS diagnosis, a physical assessment and cytogenetic testing is needed. Women with 45,X are diagnosed when they are born, mainly due to their dysmorphic characteristics and cardiovascular complications. However, in other cases the diagnosis is postponed even until adolescence, when the girls displayed absent of pubertal development, amenorrhea, and infertility [6].

Within structural chromosomal abnormalities, translocations are defined as the exchange of chromosomal segments of between two non-homologous chromosomes. There are two types of translocations: reciprocal and Robertsonian [7]. The resulting chromosomal rearrangements can be a balanced or unbalanced. Double strands breaks are prerequisites for translocations and the phenotype of unbalanced translocation carriers depends on the size, and location of the break [8].

The coexistence of autosomal translocations and structural/numerical abnormalities of the X chromosome are a rarely reported, there are 12 cases published [9–20]. Here we describe a mosaic Turner syndrome patient with a maternally inherited reciprocal translocation with breakpoints at 2p13 and 12q24.

## Case presentation

### Clinical report

**Fig. 1:**
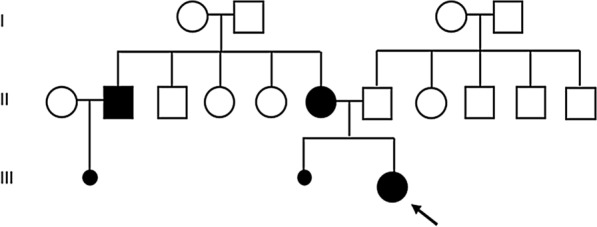
Family tree of the Turner syndrome proband carrier of the translocation (2;12)

The patient was born on July 2000 at 32 weeks of gestation after oligohydramnios diagnosis. Her measurements at birth were as follows: weight 2.4 kg (1.65 SDS), length 46.5 cm (1.53 SDS) and head circumference 31.5 cm (1.75 SDS). During the first year of life, she received endocrine therapy to enhance growth and ameliorate join pain. She was the second offspring of healthy, non-consanguineous parents, who had a previous miscarriage (Fig. 1). When she was 5 years old, an echocardiography showed she has mild tricuspid regurgitation and pulmonary arterial hypertension (41 mmHg).

**Fig. 2:**
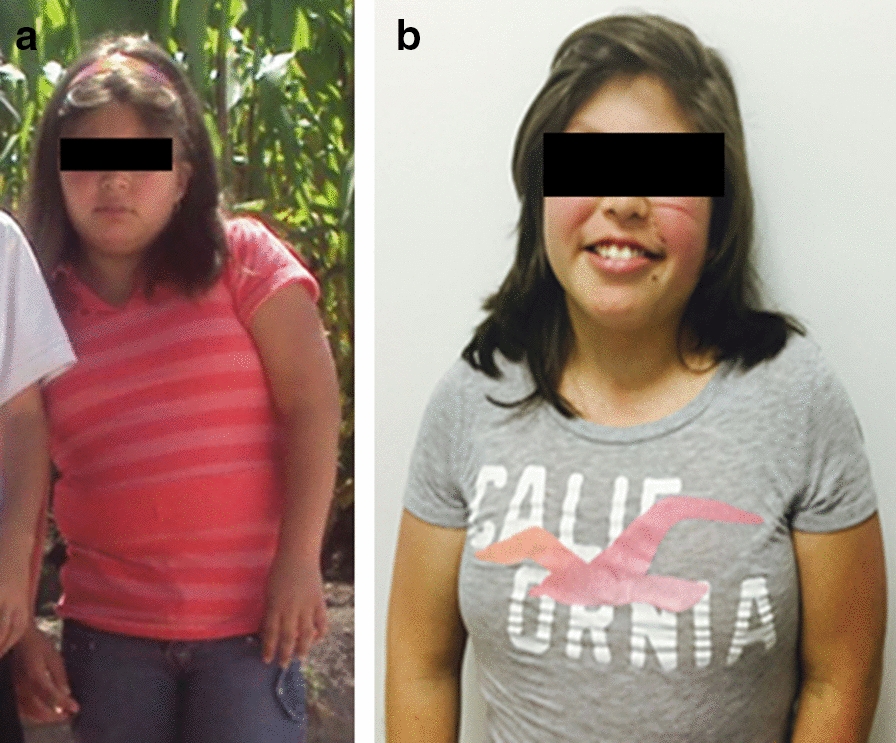
Clinical photographs of the patient. **a** At 10 years old, **b** at 16 years old

Physical examination in the first medical appointment on June 2006 evidenced low set ears, pectus excavatum, ogival palate and wide-spaced nipples (Fig. 2). Based on the symptoms and karyotype, TS was diagnosed. From 7 years of age, she has been diagnosed with myopia. The second medical appointment in 2012 confirmed the diagnosis and determined a reciprocal translocation using Giemsa trypsin G-banding (GTG). In 2015, she had thyroid disorders and was treated with levothyroxine, besides her tonsils were removed. The patient did not have cardiovascular diseases. She had mild intellectual disability. At puberty (15 years old), she showed primary amenorrhea, underdeveloped breasts, juvenile arthritis, nail dysplasia, short stature (1 m, − 9.76 SDS), and lymphedema of the feet, so she wore orthopedic insoles for walking. In the third medical examination the 16-year-old patient has an estimated 3-year delay in bone-age. The patient was on a hormone replacement therapy (patches) to stimulate sexual development. As an ultrasound scan showed no ovaries, genetic counseling was recommended to overcome future infertility complications.

### Cytogenetic and genomics assessment of proband and parents

**Fig. 3:**
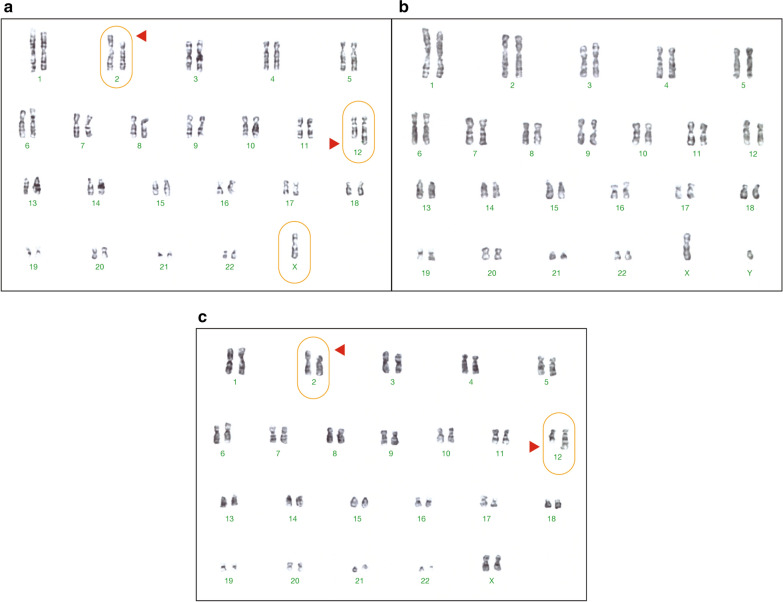
Patient and parents’ karyotypes. **a** Patient with mosaic X chromosome monosomy and t(2;12). **b** Father with normal karyotype. **c** Mother carrier of the t(2;12)

In 2006, the patient was diagnosed with a monosomy X mosaicism 45,X[90]/46,XX[10]. In 2012, with a better G-banding resolution, a reciprocal translocation with a 45,X,t(2;12)(p13;q24)[95]/46,XX,t(2;12)(p13;q24)[5] karyotype was found (Fig. 3a). To determine whether the translocation was inherited or de novo, her parents were karyotyped. The father had a normal karyotype, 46,XY (Fig. 3b), while the mother carried the reciprocal translocation 46,XX,t(2;12)(p13;q24) and transmitted it to her child (Fig. 3c).

**Fig. 4:**
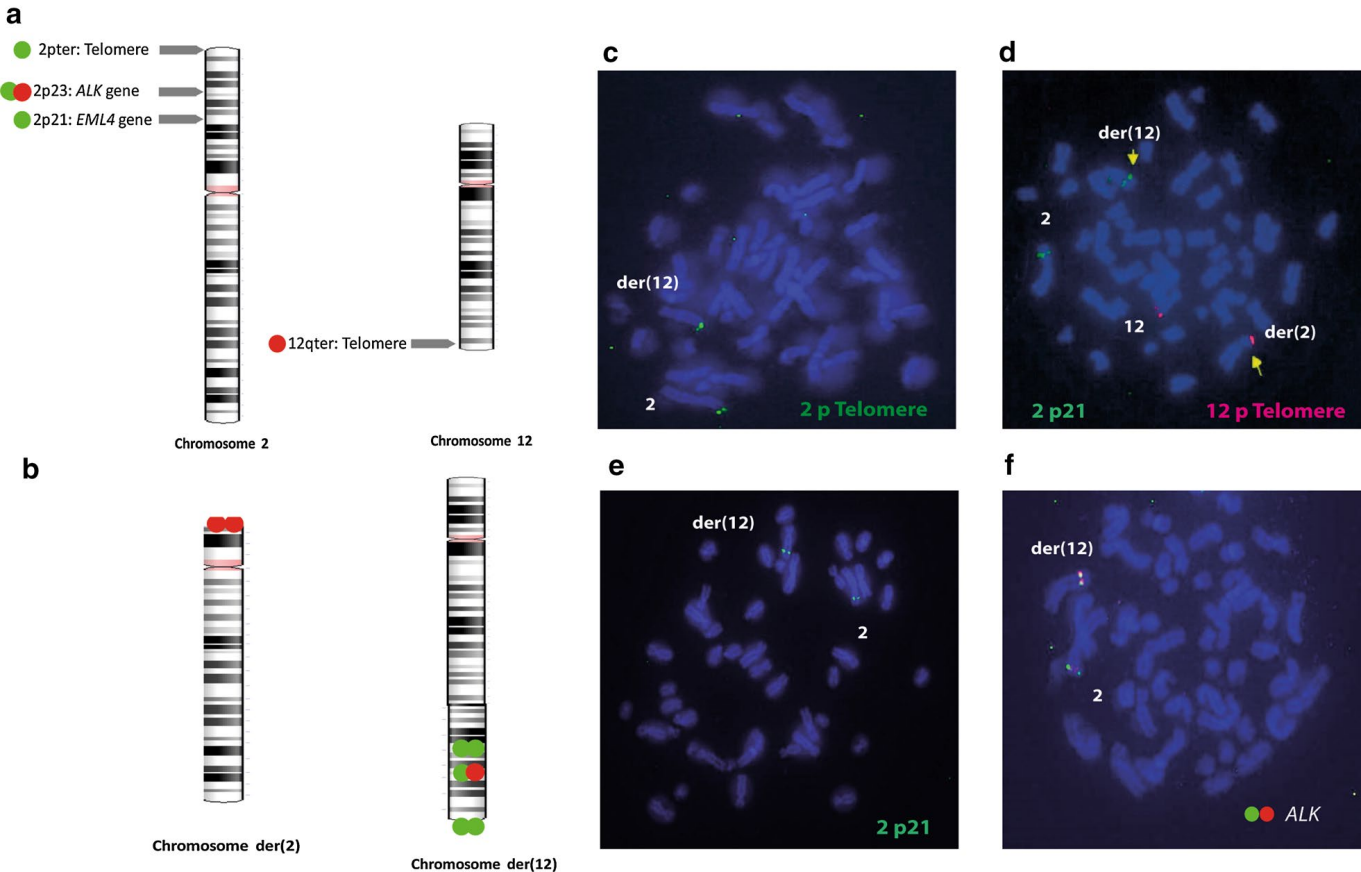
FISH on chromosomes 2 and 12 implicated in the balanced translocation. Location of FISH probes in normal chromosomes 2 y 12 (**a**) and derivatives chromosomes 2 and 12 (**b**). **c** 2p telomere (green). **d** 12q telomere (red) and 2p21 probe: *EML4* gene (green). **e** 2p21 probe (green). **f** 2p23 probe: *ALK* gene (green and read combination)

The reciprocal chromosomal exchange between chromosome 2 and 12 was visualized using fluorescence probes on the patient’s lymphocytes. Signals for the 2pter (Agilent, Santa Clara), 2p23 (*ALK*), and 2p21 (*EML4*) (Abbott Molecular, Abbott Park, IL) probes were in one normal chromosome 2 and in the derivative chromosome 12, der(12). One signal of the 12qter (Agilent, Santa Clara) was located in the telomere of chromosome 12 and in the derivative chromosome 2, der(2) (Fig. 4).

**Fig. 5:**
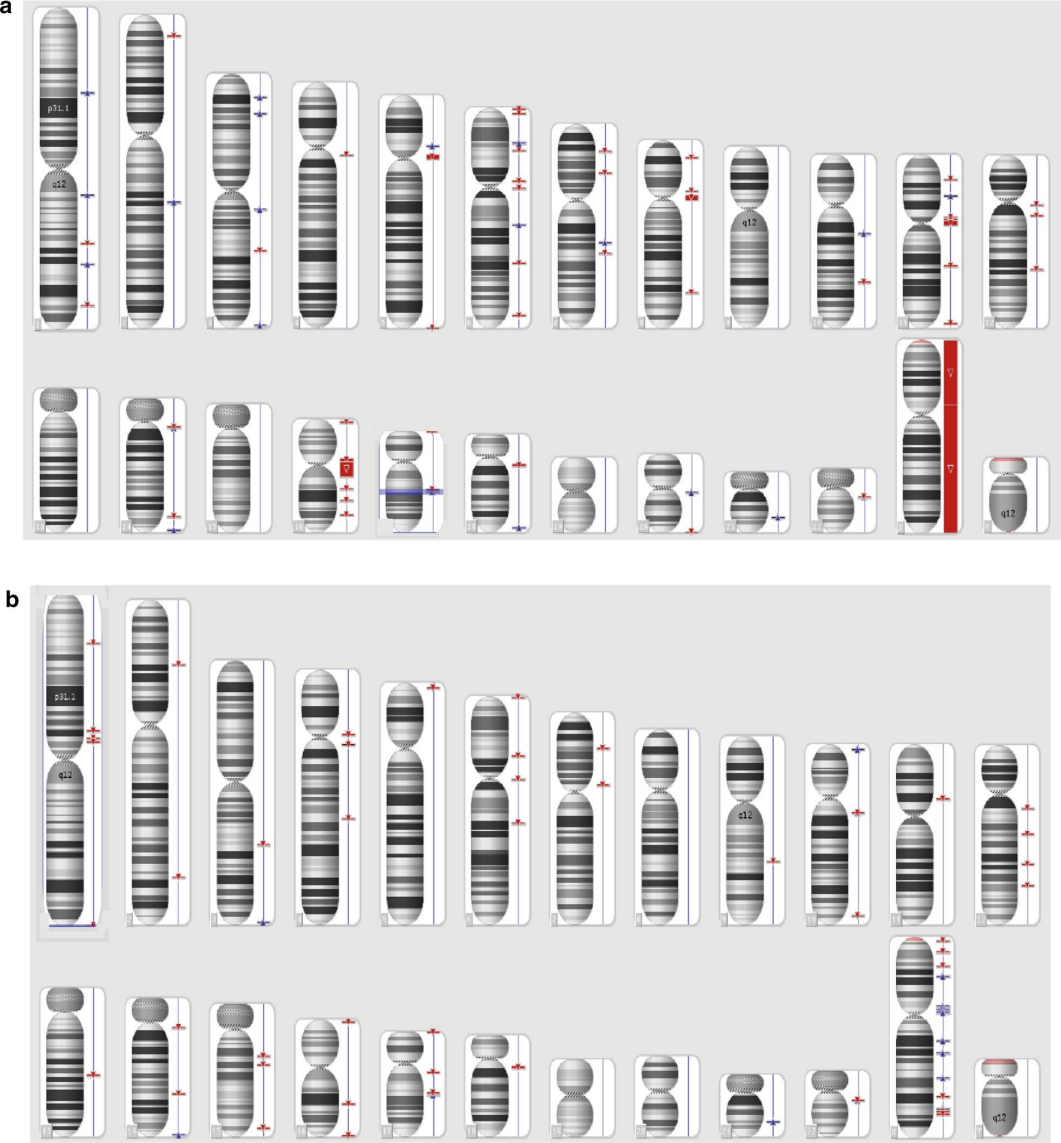
Genetic mapping array. **a** Patient. **b** Patient’s mother (blue triangle: gain; red triangle: loss)

Extracted DNA from the patient and her mother were hybridized in the CytoScan^®^ 750 K Array separately (Affymetrix, Santa Clara, CA, USA). The arrays were processed in the Fluidics Affymetrix 450 Station and scanned with GeneChip™ 3000 (Affymetrix, Santa Clara, CA, USA). The software used for the analysis was Affymetrix Chromosome Analysis Suite version 2.1. The quality control thresholds were: SNPQC ≥ 15.0; MAPD ≤ 0.25; Waviness SD ≤ 0.12. Smoothing setting was On and the Joining Option marked 25 markers, 200 kbp. The microarray data were interpreted according to the annotations of genome version “Genome browser hg19, February 2009” (GRCh37/hg19). Array results of the proband showed Copy Number Variations (CNV) in several autosomes and sex chromosomes, being the loss of the X chromosome the largest genomic alteration as indicated: arr[GRCh37] Xp22.33p11.22(168,551_53,465,323) × 1 dn,Xp11.22(53,465,326_53,477,877) × 4 mat,Xp11.22q28(53,477,878_155,233,098) × 1 dn. The 155.05-Mb loss of X chromosome involved 976 genes. In the chromosome participating in the translocation the proband have the following CNVs: arr[GRCh37] 12q11q12(38,012,530_38,385,512) × 1, 12q13.11(46,515,130_46,538,053) × 1, 12q21.32 (88,497,575_88,499,437) × 1 mat, 2p24.3 (16,062,908_16,095,671) × 1, 2p22.3 (145,196,729_145,231,557) × 3. None of them in the breakpoints, which confirmed the presence of a balanced translocation (Fig. 5a). The 3.05-Mb whole genome gain in the proband comprised 68 genes while the 184.70-Mb loss represented 1018 genes. The mother showed a total of 4.80 Mb of CNVs, with a gain of 1.70 Mb and a loss of 3.11 Mb (Fig. 5b).

**Fig. 6:**
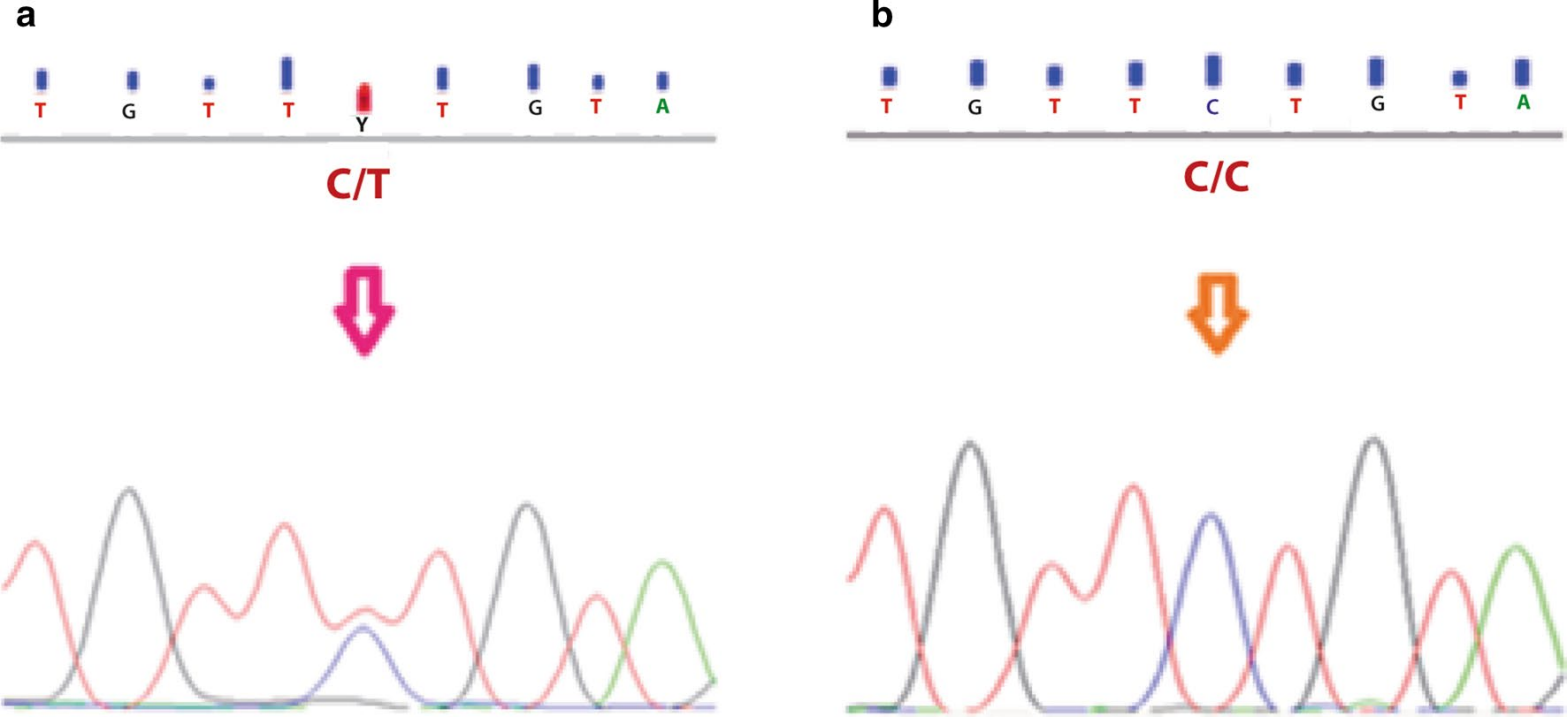
Sanger sequencing of the exon 13 of *MSH2* gene. **a** Patient. **b** Patient’s mother

The maternal genomic material displayed a loss at 2p21, involving *MSH2*. *MSH2* exon 13 along with its intronic splice-site flanking regions were amplified and sequenced using Big Dye Terminator v3.1 (Applied Biosystems, Austin, TX) in the Genetic Analyzer 3500 (Applied Biosystems, Austin, TX). Primers and PCR amplification conditions were previously reported [21]. In the targeted region the rs2303428 polymorphism was homozygous (C/C) for the mother and heterozygous for the patient (C/T) (Fig. 6).

Although the patient displayed mostly Turner phenotypic traits, she has several gain and loss regions that harbor autosomal genes. To explore the clinical features associated with these genes, a functional genomic analysis was performed in ToppFun portal based on refined list of autosomal genes (shared genes between mother and proband excluded) (Additional file 1: Table S1). Diseases associated with the refined gene list were retrieved using the following settings: false discovery rate (FDR) as the correction method and *p* value cutoff of 0.05 (Additional file 1: Table S2) [22].

## Discussion and conclusion

Monosomy of X chromosome is the most common cause of TS (50%), followed by X chromosome monosomy mosaicism (15–23%) [23]. TS females are typically shorter and have delayed puberty, as seen in our patient. Cardiovascular (50%) and kidney (33%) diseases are associated comorbidities, however our patient has overcome her cardiac complications and have never shown kidney affections [5]. Symptoms occurrence and severity vary according the percentage of 45,X/46,XX mosaicism. TS mosaic patients did not have heart surgery or take antihypertensive medication [24].

Reciprocal translocations are the most common structural with an approximate incidence of 1 in 500–625 livebirths [25]. They can be originated by non-homologous chromosomal rearrangements, exposure to genotoxic agents that damage DNA or familiar inheritance. Less than 6% of reciprocal translocations carriers have clinical features, such as autism, intellectual disability or congenital abnormalities, due to micro-deletions, duplications or intergenic losses [26]. The mother of our proband, who carried the translocation, is completely healthy and did not seek medical care [16]. In contrast, our patient search for medical assistance due to her symptoms related to TS and the reciprocal translocation discovery was an incidental finding.

The coexistence of autosomal translocations and TS cases is rarely informed. From the 12 cases published and including the case reported in this study, Robertsonian translocation between chromosome 13 and 14 was the most frequent (38.5%), followed by t(1;2) (15.4%). The other autosomal translocations t(1;9), t(2;12), t(2;22), t(4;16), t(8;19), t(15;22) were uniquely found in TS patients. In the 13 cases, 9 were inherited, 3 were de novo and the origin of one was not detected as parental karyotype was not examined. Our case is the first female with mosaic X chromosome monosomy and a reciprocal translocation involving chromosome 2 and 12. The breakpoints were determined by conventional cytogenetics on 2p13 and 12q24. FISH probes confirmed that the translocated region in chromosome 2 comprised the segment from 2p21 to 2pter, while in chromosome 12 the terminal band 12q24 was exchanged.

Considering our patient and 8 out of 12 cases with a fully described phenotype, the most frequent features were delayed postnatal growth, low posterior hairline, low seat ears, dysmorphic nails, primary amenorrhea, ogival palate, short and webbed neck, wide-spaced nipples, lymphedema of the hands and feet, normal cardiovascular parameters and renal function (Additional file 1: Table S3). The latter mentioned characteristics are typical sign of TS with no additional phenotypic effect associated with the translocations as individuals have balanced complement of genes and the translocation breakpoints mapped outside coding genes or regulatory regions [27]. Our proband displayed distinctive traits such as mild intellectual disability, myopia, pectus excavatum, thyroid problems and juvenile arthritis, so it is speculated that the genes responsible for this phenotype may be altered.

It has been suggested that translocations do not affect to the X chromosome disjunction in the gametogenesis, since it has been reported that the X chromosome in a TS patient, carrier of a maternally inherited translocation, had a material origin [17]. In other cases the reciprocal translocations and X chromosome aberrations are de novo [16, 19, 22]. Authors considered the possibility that translocations might influence non-disjunction during early stages of embryonic development [15].

In reciprocal translocations a cross-shaped quadrivalent is formed during gametogenesis and according to the chromosome segregations balanced and unbalanced gametes are produced. The resulted gametes can be grouped among four segregation modes: Alternate, Adjacent I, Adjacent II, 3:1 and 4:0. Out of the 8 possible pairs of gametes, only one pair resulted from alternate segregation, one genetically normal and the other balanced, will produce viable offspring when fertilized [28]. The rest of segregation modes will lead to imbalances. Hence it is most likely that alternate segregation enables our patient birth. In our family tree, the maternal uncle’s wife had a miscarriage, consequently it may be speculated that he is a healthy carrier of the t(2;12) translocation and that the spontaneous abortions might be a result of his unbalanced gametes fertilization.

Our group and others have found additional chromosomal changes alongside with structural chromosome aberrations, consequently genome-wide copy analysis was performed [8, 29]. After comparing whole genome CNVs in the proband versus her mother, it was found a net gain of 1.36 Mb and a net loss of 181.58 Mb. The absence of one X chromosome contributed to a large base pair loss in the proband.

Combining the genetic information and the medical record, genotype–phenotype correlation evidenced several genes responsible for the expression of Turner phenotype. For instance *SHOX* gene, located in the pseudo autosomal region of the X chromosome, is associated with idiopathic short stature and TS [30]. This is consistent with the patient abnormalities in body height that required hormonal treatment from childhood.

The prevalence of ovarian failure and visual defect might be explained as they share a similar genetic pathway. *USP9X* gene, located in X chromosome, is responsible of gonadal dysgenesis in TS cases, while in *Drosophila* it plays a role in eye and ovary development. Eye defects seemed not to be correlated with any TS karyotype or its variants [31]. Our patient has been diagnosed with myopia in her childhood and during her puberty through a pelvic echography the absence of ovaries was confirmed. Other genes such as *OPN1MW, PHEX, TEX28* are associated with X-linked myopia [22].

For the mapping array bioinformatics analysis, autosomal genes exclusively altered in the proband were evaluated. Common autosomal genes either gained or lost in the patient and her mother were not considered, as the mother was healthy and possibly her CNVs were benign. It has been reported that intellectual disability is a not a feature of TS and that only structural sex chromosome abnormalities can cause mental disturbances [32]. In this regard, our patient had a mild cognitive impairment, that might be likely related to autosomal genes. In the functional enrichment analysis, mild cognitive disorders and intellectual disability were significant terms. Besides, other medical complications mainly cancerous process were associated terms with autosomal genes in CNVs regions [22]. Thus, long-term health surveillance is highly recommended in case these malignancies have a late onset.

The amplification and sequencing of *MSH2* gene revealed the patient was heterozygous for the rs2303428 polymorphism (T/C), whereas her mother was homozygous for the mutant allele (C/C). Array results evidenced the mother had loss the 2p21 region that comprised *MSH2*. Two possibilities arise, one is that lost region have the wild type allele, in which case the mother was heterozygous or that the lost region contain the mutant allele. Based on the evidence the proband inherited the mutant allele from her mother. *MSH2* participates in the DNA repair system and correct wrong post-replicative base pair pairing. It forms a heterodimer complex with *MSH6*, *MSH3*, which binds to DNA mismatches and initiates DNA repair. *MSH2* mutations have been related to endometrial cancer and hereditary nonpolyposis colorectal cancer [32]. *MSH2*-deficiente cell lines exhibited a faulty mismatch recognition, microsatellite instability, increased recombination between homologous (non-identical regions) DNA sequence, in other words a reduction of genomic stability. Family members with *MSH2* germline mutations had somatic frameshift mutations in cellular growth, apoptosis and DNA repair genes [33]. Elevated frameshift mutations were also detected in *Mhs2* heterozygous mouse models, suggesting a haploinsufficiency of this tumor suppressor gene [34]. It can be hypothesized that maternal *MSH2* haploinsufficiency promoted genomic instability which was transmitted to her offspring.

In a family case, mother and daughter have X chromosome abnormalities and a balanced translocation with a 45,X,t(4;16)[2]/46,XX,t(4;16)[93]/47,XXX,t(4;16)[5] and 45,X,t(4;16)[9]/46,X,i(X)(q10),t(4;16)[91] karyotypes in their lymphocytes, respectively [11]. This highlighted the relevance of parental chromosome analysis of TS patients with a reciprocal, not only to identify the origin of the but also to evaluate whether parents carry numerical X chromosome abnormalities in a mosaic form. Normal fertility in women with 45,X could be explained by the presence of an additional normal cell line in their gonads or the absence of gonadal mosaicism. Annerén et al. [18] reported that an elder mosaic Turner’s syndrome woman, with a familial reciprocal translocation detected in lymphocytes and fibroblast, delivered 3 normally developed children and had not prior history of abortion. The latter cases showed family planning genetic counselling should be performed in a case-by-case basis.

All in all, based on patient’s symptoms and the presence of mosaic karyotype with one cell line with X chromosome loss, TS was diagnosed. Additionally, a maternally inherited reciprocal translocation was noted between chromosome 2 and 12. Possibly the maternal side of the family carried the balanced translocation as recurrent miscarriages are seen in the family tree. Through genetic mapping arrays analysis, a genotype–phenotype correlation could be performed. Patient symptoms are closely related to the loss of homologous sex chromosome. The combination of cytogenetics, and molecular biology techniques provided a comprehensive analysis of the patient genome alterations that aids genetic counseling.

## Supplementary information

**Additional file 1.** ST1 (sheet 1): Supplementary Table 1: Genes implicated in the region loss or gain; ST2 (sheet 2): Supplementary Table 2: Human Diseases associated with 87 (out of 89) autosomal genes found exclusively in the proband, and ST3 (sheet 3): Supplementary Table 3: Clinical findings of Turner Syndrome patients’ carriers of balanced translocations including our case.

## Data Availability

All data is available from the corresponding author on reasonable request.

## Abbreviations

TS: Turner Syndrome
kg: Kilograms
cm: Centimeters
m: Meters
SDS: Standard deviation score
GTG: Giemsa trypsin G-banding
ALK: Anaplastic lymphoma kinase
EML4: Echinoderm microtubule-associated protein-like 4
SNPQC: SNP array quality control
MAPD: Median of the absolute values of all pairwise differences
kbp: Kilobase pair
CNV: Copy number variation
GRCh37: Genome reference consortium human genome build 37
Mb: Mega base pairs
MSH2: MutS homolog 2
USP9X: Ubiquitin specific peptidase 9 X-linked
OPN1MW: Opsin 1 medium wave sensitive
PHEX: Phosphate-regulating neutral endopeptidase
TEX28: Testis expressed 28
